# The inducible gametocyte producer (iGP1) strain is well-suited to produce both immature and mature gametocytes for subsequent drug sensitivity profiling

**DOI:** 10.3389/fcimb.2026.1809263

**Published:** 2026-07-01

**Authors:** Thomas Martin Schäfer, Lais Pessanha de Carvalho, Fatih D. Darende, Daniel Stopper, Finn K. Hansen, Jana Held

**Affiliations:** 1 Institute of Tropical Medicine, University of Tübingen, Tübingen, Germany; 2 Department of Pharmaceutical and Cell Biological Chemistry, Pharmaceutical Institute, University of Bonn, Bonn, Germany; 3 German Center for Infection Research (DZIF), Tübingen, Germany; 4 Centre de Recherches Médicales de Lambaréné, Lambaréné, Gabon

**Keywords:** drug sensitivity assay, dual-active antimalarials, gametocytes, iGP1, *Plasmodium falciparum*

## Abstract

Advancing toward malaria eradication requires compounds that target both asexual blood-stage parasites to treat the disease as well as gametocytes to reduce transmission. However, current methods for gametocyte production and subsequent drug sensitivity testing, especially for immature gametocytes, lack standardization and are labor-intensive. Here, we describe a simplified and reproducible workflow for assessing gametocytocidal activity using the inducible gametocyte producer 1 (iGP1) line combined with the luciferase-based BacTiter-GLO viability detection kit. This approach yielded highly synchronous immature gametocytes enabling a robust drug sensitivity assay (Z´ = 0.65 ± 0.25). Testing of established and novel antimalarial compounds against immature and mature iGP1-gametocytes showed activity profiles comparable to previously published results, validating our approach. Of note, chlorotonil A and boromycin showed potent low-nanomolar activity against both immature and mature gametocytes, while the histone deacetylase inhibitors DS-089 and DS-118 displayed moderate (sub-) micromolar activity. In summary, this workflow provides a reliable and user-friendly platform for the generation of immature and mature gametocytes and subsequent drug sensitivity profiling with the potential to facilitate the discovery of novel gametocytocidal compounds and dual-active antimalarial treatments.

## 1 Introduction

To this day, malaria has been one of the most devastating infectious diseases. In 2024 alone, the World Health Organization (WHO) estimated a number of 282 million cases, resulting in 610,000 fatalities (WHO, 2025). The majority of the disease burden is located in Sub-Saharan Africa and can be attributed to infections with *Plasmodium falciparum* (WHO, 2025). Furthermore, the impact of malaria on global health will likely increase in the wake of the ongoing climate change, with higher temperatures, humidity and extreme weather events like flooding promoting mosquito development, viability and feeding activity as well as plasmodial infection and propagation (Megersa and Luo, 2025; Symons et al., 2026). The life cycle of *P. falciparum* involves humans as intermediate and various species of anopheline mosquitoes as final hosts (Fikadu and Ashenafi, 2023). While malaria symptoms are caused by asexual replication in the host’s erythrocytes, a fraction of asexual parasites commits to sexual differentiation into the transmissible gametocytes, in both a stochastic manner as well as due to various external stressors (Fikadu and Ashenafi, 2023; Harris et al., 2023; Voss and Brancucci, 2024). In most cases, parasites follow the route of next cycle commitment, meaning that ring-stages will become gametocytes one asexual cycle after the commitment (Bancells et al., 2019). On a molecular level, sexual commitment is driven by the transcription factor *Pf*AP2-G, which is epigenetically repressed by trifold methylation of the lysine 9-residue of histone 3 and binding of heterochromatin protein 1 (*Pf*HP1) to the co-localized DNA, making the gene inaccessible to the parasitic transcription machinery (Flueck et al., 2009; Brancucci et al., 2014). This process can be reversed by the expression of the protein gametocyte development 1 (GDV1), which directly binds to P*f*HP1 and removes it from the *pfap2-g* locus (Filarsky et al., 2018). Gametocyte development of *P. falciparum* typically lasts between 10 to 12 days and involves five morphologically distinct stages (Ngotho et al., 2019). Stages I-IV are sequestered in the bone marrow, while the transmissive stage V gametocytes are released into the bloodstream to be taken up by a mosquito, thus completing the parasite’s life cycle (Ngotho et al., 2019).

The current gold standard treatment, artemisinin-based combination therapies (ACTs), mainly target asexual blood-stage parasites, and to a limited degree immature gametocytes (Plouffe et al., 2016; Chutiyami et al., 2024). To reduce gametocyte carriage WHO recommends adding a single low dose primaquine (0.25 mg/kg) to ACT-regimens (WHO, 2024), balancing transmission reduction and the risk of haemolysis linked to glucose-6-phosphate dehydrogenase deficiency (Yilma et al., 2023, 2025). However, uptake of this recommendation in Africa remains limited (Mwaiswelo et al., 2022). In addition, artemisinin partial resistance in Africa is posing a threat for successful antimalarial treatment (Conrad et al., 2023; Kamya et al., 2025). Targeting the transmissible gametocytes is essential to curb the spread of drug-resistant parasites and to advance malaria elimination. Therefore, the development of novel treatment combinations targeting the symptom-causing asexual parasites as well as transmissible gametocytes is of utmost importance (Burrows et al., 2017; Schäfer et al., 2024).

To identify novel gametocytocidal compounds, robust and reproducible *in vitro* screening platforms are needed. Currently, this is complicated by several factors. Sexual conversion is commonly induced by various stress conditions, e.g. the use of conditioned medium or choline depletion, high parasitaemia or sudden drop in haematocrit (Omorou et al., 2022). This necessitates rigorous synchronization of asexual parasites and a strategy to allow well-timed induction of sexual commitment to achieve highly synchronized gametocyte cultures, making the cell culture maintenance work- and cost-intensive (Plouffe et al., 2016; Reader et al., 2022). A recent innovation to simplify gametocyte culturing techniques is the inducible gametocyte producer 1 (iGP1)-strain, which was genetically engineered to conditionally express GDV1 via two regulatory elements (Boltryk et al., 2021). On the transcript level, the *glmS*-ribozyme self-cleaves the transgenic transcript in the presence of D-glucosamine, while on the protein level a destabilization domain targets the transgenic GDV1 for proteasomal degradation in the absence of the stabilizing ligand Shield-1 (Boltryk et al., 2021). Removal of D-glucosamine and simultaneous addition of Shield-1 therefore results in a coordinated induction of sexual commitment and subsequently in a synchronously developing population of gametocytes (Boltryk et al., 2021).

The main goal of this study was to develop a straightforward workflow for the drug sensitivity profiling of antimalarial compounds against immature and mature gametocytes. To do so, we assessed whether the iGP1-line allows for a simplified gametocyte production for subsequent drug sensitivity assays. For immature gametocytes, we compared synchronicity of iGP1-cultures to conventional methods and describe and validate a microdilution drug sensitivity assay. For mature gametocytes, we used the identical assay and compared drug sensitivity profiles of iGP1 and conventionally produced gametocytes.

## 2 Material and methods

### 2.1 Continuous culture of asexual parasites

The iGP1 was kindly provided by Till Voss from the Swiss Tropical and Public Health Institute. The laboratory strain NF54 (a vigorous gametocyte producer) was initially acquired from Sanaria, Inc in the form of cryopreserved sporozoites and asexual blood-stage parasites were later isolated from an infected participant of a clinical trial (Mordmuller et al., 2015). For parasite propagation, O^+^ erythrocytes and human AB-serum were obtained from the blood bank of the University Hospital of Tübingen, Germany.

Asexual parasites were cultured as described previously with modifications (Trager and Jensen, 1997). Briefly, parasites were grown in RPMI-1640 medium supplemented with final concentrations of 0.45% (w/v) AlbuMax, 25 mM HEPES, 2 mM L-glutamine, 20 mg/L hypoxanthine, and 50 µg/ml gentamicin (complete medium). If not stated otherwise, cultures were always kept at 2.5% haematocrit and below 3% parasitaemia. For iGP1, D-glucosamine hydrochloride (Sigma-Aldrich, USA) was added to a final concentration of 2.5 mM to prevent induction of GDV1-expression. Medium was replaced three times per week, and the culture was diluted with fresh O^+^ erythrocytes when necessary. Parasitaemia was assessed using thin blood smears that were fixed in absolute methanol for 10 seconds and stained using Hemacolor (Merck, Germany, 1.11661) or 5% Giemsa (Merck, Germany, 1.09203) in phosphate buffer according to the manufacturer’s instructions. If necessary, parasites were synchronized using sorbitol by taking them up in 10x the erythrocyte volume of 5% D-sorbitol, incubating for 5 minutes and washing with 10 ml culture medium.

### 2.2 Differentiation of immature stage II/III iGP1 gametocytes

**Figure 1:**
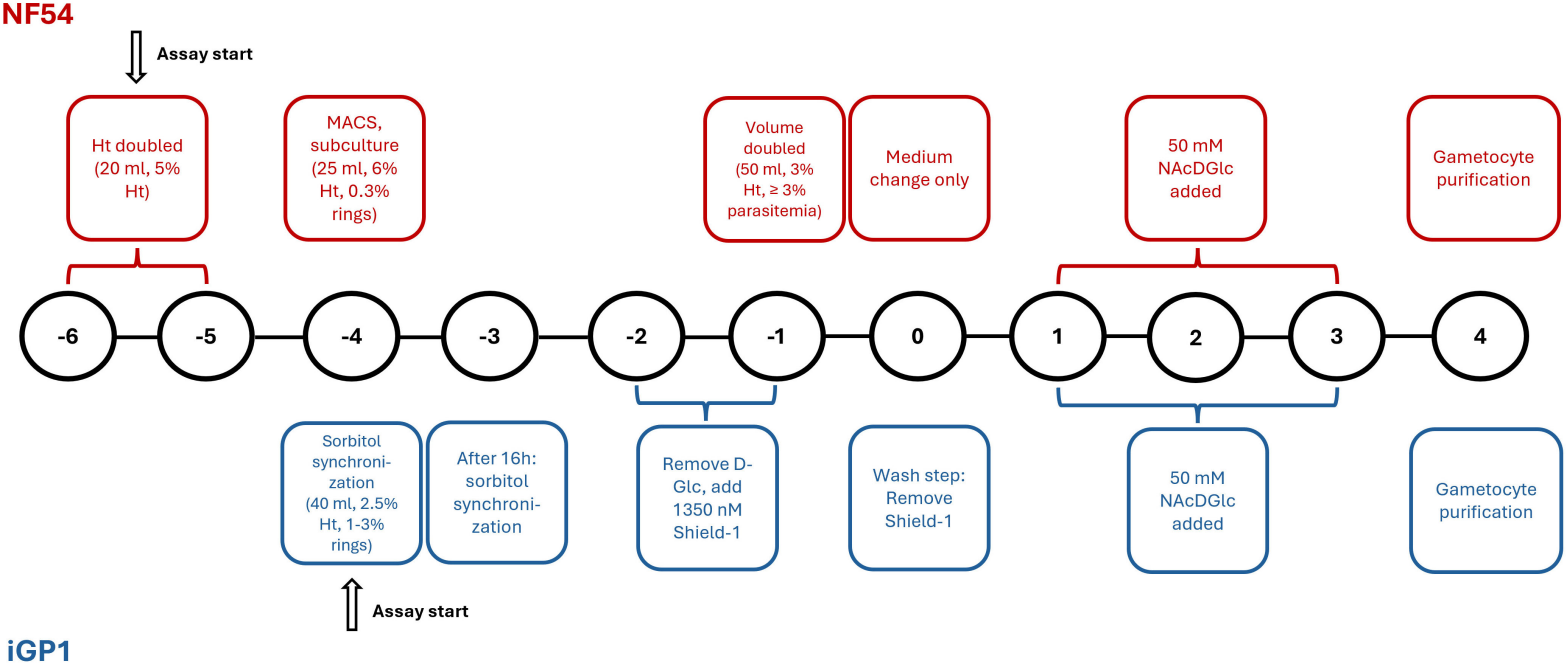
Timeline depicting the experimental procedures to generate immature stage II/III gametocytes using the strains NF54 and iGP1. Cultures were kept at the indicated haematocrit, parasitaemia and total culture volume. The numbers correspond to assay days and, in the case of positive numbers, also to the days of gametocyte development. Day 0 marks the day of gametocyte induction. Ht, haematocrit; MACS, magnetic-assisted cell sorting; D-Glc, D-glucosamine; NAcDGlc, N-acetyl-D-glucosamine hydrochloride.

To assess synchronicity and for subsequent drug sensitivity testing, immature (stage II/III) iGP1-gametocytes were produced as described previously with minor modifications (Boltryk et al., 2021) (see **Figure 1** for graphical explanation of the protocol). The total protocol takes 9 days: starting on day -4 until purification on day 4. In detail, asexual parasite cultures (2.5% haematocrit, 1-3% parasitaemia, total culture volume 40 ml) were synchronized using 5% D-sorbitol two times in an interval of 16 hours as described above (D-4 and D-3), followed by an incubation time of 32 hours. Induction was then performed with young ring-stages (0–8 hours post invasion) at D-2 by washing parasites with complete medium without D-glucosamine and taking them up in induction medium consisting of complete medium with 5% (v/v) heat-inactivated human AB serum and 1350 nM Shield-1 (MW = 748.9, MedChemExpress, USA, HY-112210). On D-1, induction medium was replaced. From D0 onwards, complete medium with 5% (v/v) heat-inactivated human AB-serum (gametocyte medium) was added to the cultures instead of induction medium and medium was replaced daily. On D4, a thin blood smear was prepared to assess healthy gametocyte morphology (Saliba and Jacobs-Lorena, 2013) and presence of primarily immature stage II/III gametocytes. On the same day, the gametocytes were purified using magnetic-activated cell sorting (MACS) with LD-columns (Miltenyi Biotec, Germany, 130-042-901) and counted using a Neubauer counting chamber for subsequent drug sensitivity testing as previously described (de Carvalho et al., 2021).

### 2.3 Differentiation of immature stage II/III NF54 gametocytes

To compare the synchronicity of iGP1 immature gametocyte cultures to a conventional method, immature NF54 gametocytes were differentiated using the previously described “crash”-method with modifications (Gebru et al., 2017) (see **Figure 1**). The total protocol takes 11 days: starting on day -6 until purification on day 4. On day -6, the haematocrit of the NF54 asexual culture was doubled to 5% (20 ml total culture volume). On D-4, the asexual culture was synchronized using MACS with LD-columns. The flowthrough containing the ring-stage parasites was collected and a subculture was prepared at 6% haematocrit and 0.3% parasitaemia in gametocyte medium (total culture volume of 25 ml). From D-3 onwards, gametocyte medium was changed daily without parasite dilution. On D-1, the haematocrit was dropped to 3% by doubling the volume of gametocyte medium (total culture volume of 50 ml). From D1-D3, N-acetyl-D-glucosamine hydrochloride was added to a final concentration of 50 mM during medium replacement to eliminate remaining asexual parasites. On D4, the immature gametocytes were processed as described above for iGP1 (de Carvalho et al., 2021).

### 2.4 Differentiation of mature stage IV/V iGP1 gametocytes

**Figure 2:**
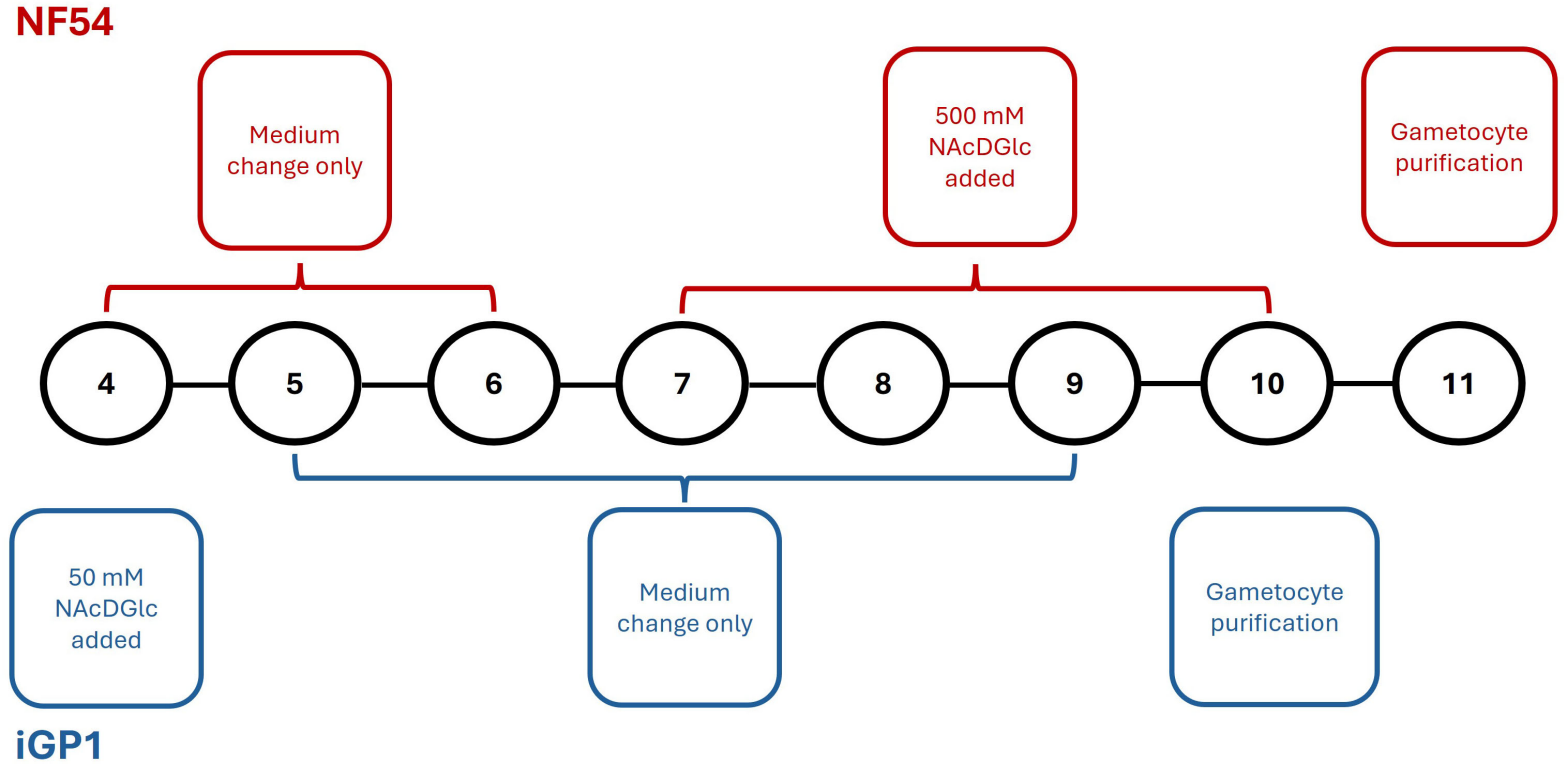
Timeline depicting the experimental procedures to generate mature stage IV/V gametocytes using the strains NF54 and iGP1. The numbers correspond to assay days and, in the case of positive numbers, also to the days of gametocyte development. Assay timelines from Day -6 to Day 3 (for NF54) and -4 to Day 3 for iGP1 are identical as for the immature gametocytes. Ht, haematocrit; MACS, magnetic-assisted cell sorting; D-Glc, D-glucosamine; NAcDGlc, N-acetyl-D-glucosamine hydrochloride.

The protocol to differentiate mature stage IV/V iGP1 gametocytes has a duration of 15 days: starting on day -4 until day 10 (see **Figures 1**, **2**). Parasites were treated identically to immature stage II/III gametocytes until D4, with two sorbitol synchronizations in a 16-hour interval and induction of sexual commitment by removing D-glucosamine and adding Shield-1 (see **Figure 1**). From D0 onwards, the cultures received RPMI-1640 medium supplemented with final concentrations of 0.8% (w/v) AlbuMax, 25 mM HEPES, 2 mM L-glutamine, 20 mg/L hypoxanthine, 50 µg/ml gentamicin and 5% (v/v) heat-inactivated human AB-serum. Medium was replaced daily until D10 (see **Figure 2**), when mature gametocytes were purified using MACS with LD-columns and density gradient centrifugation using 27.6% NycoDenz (MW = 821 g/mol, Serumwerk Bernburg, 18003) and centrifugation with 800g for 20 min at low deceleration (de Carvalho et al., 2021).

### 2.5 Differentiation of mature stage IV/V NF54 gametocytes

To compare drug sensitivity of iGP1 and conventionally produced mature stage IV/V gametocytes, NF54 cultures were treated as described for immature gametocytes with modifications including the use of RPMI-1640 medium supplemented with final concentrations of 0.8% (w/v) AlbuMax, 25 mM HEPES, 2 mM L-glutamine, 20 mg/L hypoxanthine, 50 µg/ml gentamicin and 5% (v/v) heat-inactivated human AB-serum. The total protocol duration to produce mature gametocytes of NF54 is 18 days: starting on day -6 until day 11 (see **Figures 1**, **2**). Subcultures (6% haematocrit, 0.3% ring-stage parasitaemia, total culture volume of 25 ml) were prepared as described for immature stage II/III NF54 gametocytes with induction through drop in haematocrit on D-1. However, 50 mM N-acetyl-D-glucosamine hydrochloride was not added early on to remove asexual parasites, but for four consecutive days before gametocyte purification (D7-D10) (see **Figure 2**). On D11, the mature gametocytes (stage IV/V) were purified and quantified as described for iGP1 and used for subsequent drug sensitivity testing (de Carvalho et al., 2021).

### 2.6 Staging of immature gametocyte cultures

On the day of purification, stained thin blood smears were prepared, and the number of gametocytes was counted. For every thin blood smear, at least 3000 erythrocytes were counted. The developmental stages of gametocytes were determined morphologically in accordance with literature (Saliba and Jacobs-Lorena, 2013) by an experienced microscopist. Stage II and III-gametocytes were considered as immature, while stage IV- and V-gametocytes were considered mature.

### 2.7 Drug sensitivity testing for asexual blood-stage parasites

**Figure 3:**
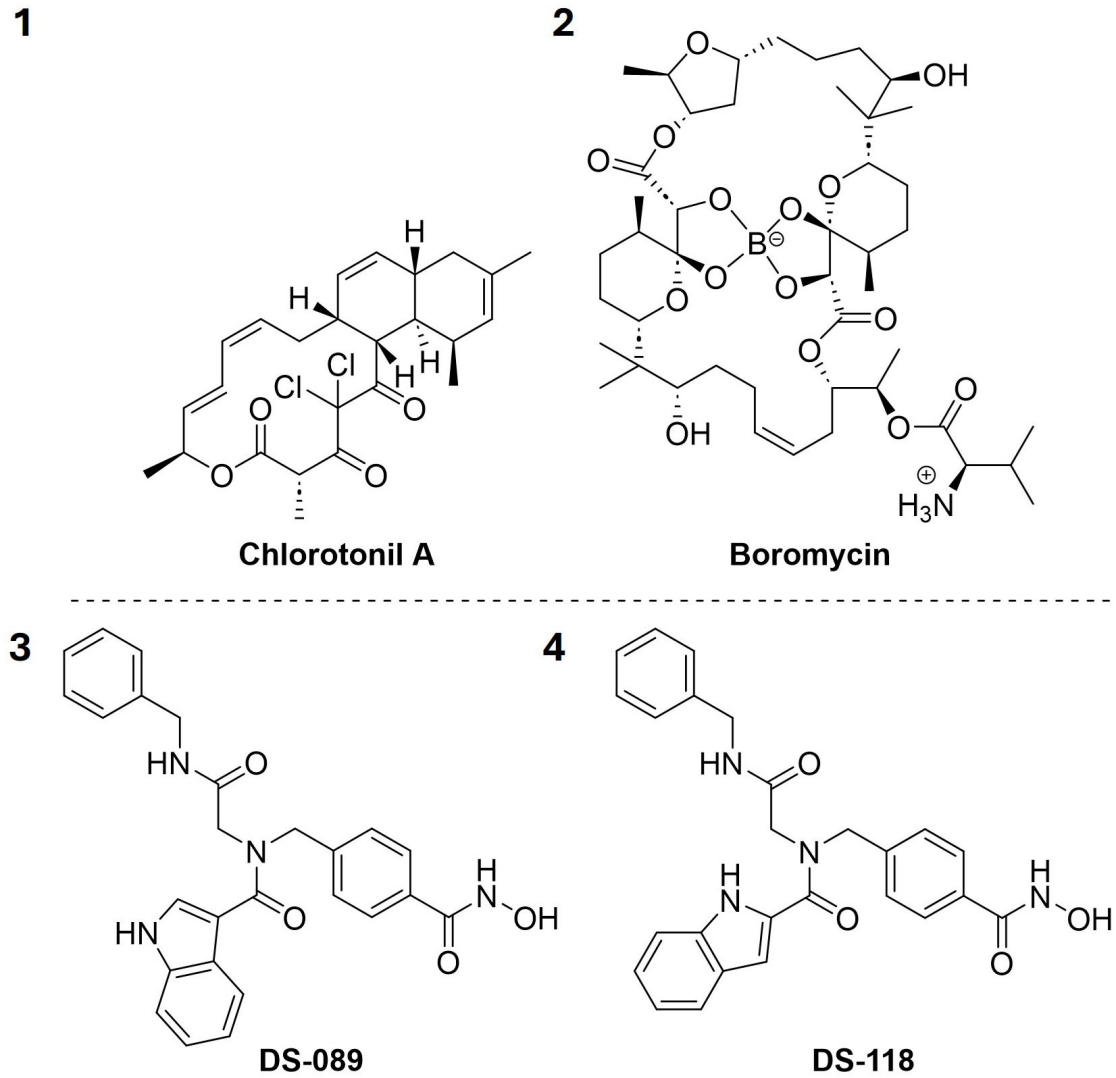
Chemical structures of the novel antimalarial candidate compounds chlorotonil A and boromycin (natural products) and the synthetic histone deacetylase inhibitors (HDACi) DS-089 and DS-118.

For drug sensitivity testing, the following compounds were used: The approved antimalarial drugs artesunate (MW = 384.42g/mol, Sigma-Aldrich, USA, A3731), chloroquine diphosphate (MW = 515.86 g/mol, Sigma-Aldrich, USA, C6628), atovaquone (MW = 366.84 g/mol, Sigma-Aldrich, USA, PHR1591), proguanil hydrochloride (MW = 290.20 g/mol, Jacobus Pharmaceuticals, USA); ferroquine (MW = 433.8 g/mol, Sanofi Synthélabo, France) and ZY19489 (MW = 465.57 g/mol, Zydus Lifesciences, India) which are currently under clinical development; the known gametocytocidal controls methylene blue (MW = 319.85, Sigma-Aldrich, USA, M9140) and epoxomicin (MW = 554.72, MedChemExpress, USA, HY-13821) as well as the novel antimalarial candidates (see **Figure 3**) chlorotonil A [MW = 479.4 g/mol, kindly provided by the Helmholtz Center for Pharmaceutical Research Saarbrücken, Germany (Held et al., 2014)], boromycin (MW = 879.87 g/mol, Santa Cruz Biotechnology, USA [de Carvalho et al., 2021)], DS-089 (MW = 456.5 g/mol) and DS-118 [MW = 456.5 g/mol, both kindly provided by Finn K. Hansen, University of Bonn, Germany (Stopper et al., 2024)]. Stock solutions of the compounds were either prepared in distilled water (methylene blue and chloroquine) or DMSO (all other compounds). Working solutions were further diluted in complete medium for NF54 and complete medium with 2.5 mM D-glucosamine for iGP1 to the desired concentration with a final dilution of at least 1:900 to prevent solvent toxicity. All tested drug concentrations can be found in **Supplementary Table 1**.

Flat-bottom 96-well plates were coated with the compounds in a 1:3 serial dilution, including 11 dilution wells and 1 well for a drug-free parasite growth control. Ring-stage parasites of iGP1 and NF54 were isolated using MACS with LD-columns and added to the wells with a final haematocrit of 1.5% and a final parasitaemia of 0.05%. The plates were incubated for 72h at 37 °C, 5% O_2_ and 5% CO_2_ before being frozen and thawed 3 times for haemolysis.

Parasite growth was determined using a histidine rich protein 2 (HRP2)-ELISA as described previously (Noedl et al., 2005; de Carvalho et al., 2019). Briefly, high-binding 96-well plates were coated with 1 µg/ml mouse IgM anti-HRP2 antibody (MPFM-55A, ICS, USA) overnight. On the next day, plates were blocked with PBS + 2% (w/v) bovine serum albumin (Fraction V, Serva, Germany, 11930.4) for 2h at room temperature. Plates were then washed 3 times with PBS + 0.05% Tween-20 (Sigma-Aldrich, USA, P1379), banged dry, wrapped in Parafilm and frozen at -20 °C until use. Afterwards, samples were diluted 1:1 with deionized water, added to the plate and incubated for 1h at room temperature. The plates were then washed 3x with PBS + 0.05% Tween-20 and 0.05 µg/ml mouse IgG HRP-coupled anti-HRP2 antibody (MPFG-55P, ICS) was added in PBS + 2% BSA + 1% Tween-20 and incubated for 1h at room temperature. Finally, the plates were washed with 3x with PBS + 0.05% Tween-20 and TMB-One substrate (Kementec, Denmark) was added and incubated in the dark for 5–10 minutes. The colour reaction was stopped by adding 1M HCl and a colorimetric measurement (450 nm) was immediately performed using a ClarioSTAR microplate reader (BMG Labtech, Germany).

### 2.8 Drug sensitivity testing for immature and mature gametocytes

Working solutions of the compounds described above were prepared using complete medium. Flat-bottom 96 well plates were coated with the drugs with a 1:3 serial dilution, including 7 dilution wells and 1 drug-free parasite growth control well. Subsequently, 50,000 gametocytes were seeded in each well. Gametocytes were incubated with the compounds for 48 hours at 37 °C, 5% O_2_ and 5% CO_2_. Afterwards, we tested two different readout methods that have previously been used for gametocyte drug sensitivity assays, namely AlamarBlue, a viability-specific resazurin dye (Reader et al., 2015), and the BacTiter-GLO kit, using ATP-production as a proxy for parasite viability (Lelievre et al., 2012). For Alamar Blue, 10 µl 10x solution were added per well after 24h of incubation and fluorescence (540–570 nm excitation, 580–610 nm emission) was determined after another 24h using a ClarioSTAR microplate reader (BMG Labtech, Germany). For BacTiter-GLO, 30 µl of each sample were transferred to a white 96-well half-area plate and mixed with 30 µl BacTiter-GLO solution. Luminescence was immediately measured using a ClarioSTAR microplate reader (BMG Labtech, Germany). Since we only found interpretable results for the measurements with BacTiter-GLO, this detection method was used throughout this study.

Several incubation times were tested to determine optimal conditions for the readout with the BacTiter-GLO kit: (A) 48h incubation with compounds, (B) 24h incubation with compounds and 24h incubation with drug-free medium, (C) 47.5h incubation with compounds and 0.5h incubation with drug-free medium and (D) 72h incubation with compounds, all at 37 °C, 5% O_2_ and 5% CO_2_. Condition A yielded the most consistent results compared to conditions B and C, while condition D drastically reduced viability of untreated gametocytes. Therefore, an incubation time of 48 hours at 37 °C, 5% O_2_ and 5% CO_2_ was used for all reported drug sensitivity assays. Mature gametocytes were incubated under the same conditions, and viability was determined using the BacTiter-GLO kit as previously described (Lelievre et al., 2012). Quality control for each assay included (i) confirmation of stage-specific gametocyte morphology, (ii) absence of significant border effects, (iii) successful activity of at least two gametocytocidal controls (methylene blue, epoxomicin, chlorotonil A), (iv) sigmoidal dose–response curves, and (v) absence of statistical outliers; only assays/values meeting all criteria were included.

### 2.9 Exflagellation assay with mature iGP1 gametocytes

To verify the quality of the generated iGP1-gametocytes, gametocyte cultures at D10 were used for an exflagellation assay. First, a stained thin blood smear was prepared to determine the gametocytaemia by counting at least 3000 erythrocytes. Next, 2 ml of gametocyte culture was centrifuged (800g, 1 min) and the supernatant discarded. The pellet was then taken up in 200 µl complete medium or complete medium with 10 µM methylene blue and 100 µl per well were added to a flat-bottom 96-well plate and incubated for 48 hours at 37 °C, 5% O_2_ and 5% CO_2_. Next, the culture was transferred to a 1.5 ml Eppendorf cup and centrifuged to remove the supernatant. The pellet was taken up in the same volume of heat-inactivated human AB-serum and incubated for 25 minutes in a cooling block at 18 °C. The sample was then diluted 1:10 with serum and transferred into an improved Neubauer counting chamber. Cells were allowed to settle down and the slide was observed under 40x magnification for rapid movement and cell aggregation, indicative of exflagellation centers. The number of erythrocytes was also determined using the Neubauer counting chamber with a 1:500 dilution in complete medium. Results were reported as exflagellation centers per µl and 0.1% gametocytaemia of the initial culture and as exflagellation centers/1000 gametocytes.

### 2.10 Determination of assay parameters

To evaluate the linear dynamic range of the assay, 1:2 serial dilutions of immature iGP1-gametocytes (ranging from 100,000 - 1,562.5 parasites per well and one well with medium-only) were prepared in sterile 96-well flat-bottom plates and incubated for 48 hours at 37 °C, 5% O_2_ and 5% CO_2_. Afterwards, luminescence was measured using the BacTiter-GLO kit according to the manufacturer’s instructions. Mean and standard deviation were calculated for 5 biological replicates with at least two technical replicates and a linear regression was conducted.

The robustness of the assay was assessed using the Z´-factor. To do so, 50,000 untreated gametocytes were used as negative control (no inhibition) and 50,000 gametocytes treated for 48h with the highest concentration (either 3.75 µM or 4.33 µM) of chlorotonil A, a potent gametocytocidal compound (Held et al., 2014), were used as positive control (maximum inhibition). Means and standard deviations were calculated for 5 biological replicates with at least two technical replicates. Afterwards, the Z´-factor was calculated as described previously (Zhang et al., 1999):


$$Z^{\prime }=1-\frac{3\times (\sigma_{positive}+\sigma_{negative})}{|{µ}_{positive}-{µ}_{negative}|\;}$$

with µ = mean and σ = standard deviation of the positive and negative controls.

### 2.11 Statistical evaluation

The IC_50_-values of the tested compounds were calculated using the drc-package version 3.0–1 with R version 4.3.2. Descriptive statistics were performed using Microsoft Excel version 1808 or GraphPad PRISM version 8.4.3. Data visualizations and linear regression were performed using GraphPad PRISM version 8.4.3 and 11.0.1. Statistical outliers were removed using Dixon’s Q with 95% confidence level (Dean and Dixon, 1951), and critical values to compare with the calculated Dixon’s Q were taken from (Verma and Quiroz-Ruiz, 2018).

## 3 Results

### 3.1 iGP1 strain is superior to NF54 in producing synchronous populations of immature stage II/III gametocytes

**Figure 4:**
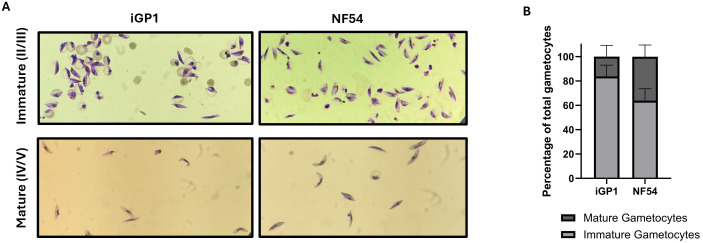
Stage-specific production of gametocytes using the *P. falciparum* strains iGP1 and NF54. **(A)** Micrographs of Hemacolor- or Giemsa-stained thin smears of iGP1 and NF54 gametocyte cultures after purification at D4 for immature stage II/III or D10 for mature stage IV/V gametocytes, respectively (light microscopy using 100x magnification with oil immersion). **(B)** Comparison of the fraction of immature stage II/III gametocytes on D4 generated using NF54 and iGP1 (3 biological replicates for NF54, 4 biological replicates for iGP1).

Our first aim was to assess the synchronicity of gametocyte development of the iGP1-strain in comparison to the NF54-strain and to previously published results (Boltryk et al., 2021; Brancucci et al., 2025). For this purpose, sexual commitment was induced in synchronized ring-stages of both strains, either via the environmental stressors of high haematocrit (6%) and high parasitaemia (3%) (NF54) followed by haematocrit drop or through timed expression of GDV1 and subsequently AP2-G through removal of D-glucosamine and addition of Shield-1 as described above (iGP1). The gametocyte morphology and stages were then examined on day 4 of gametocyte maturation (D4) (for immature gametocytes) and D10 (for mature gametocytes) using light microscopy (**Figure 4**). On D4, NF54 cultures consisted of 63.8 ± 9.7% stage II/III immature gametocytes and 36 ± 9.6% mature stage IV gametocytes. In contrast, iGP1-cultures at D4 were more synchronous, consisting of 83.8 ± 9.2% stage II/III immature gametocytes and 16.3 ± 9.2% stage IV mature gametocytes. Both strains showed the classically described morphology: Stage II gametocytes showed an oval-shaped form, III gametocytes exhibited an intraerythrocytic, half-round morphology with one flattened side and diffuse chromatin, while stage IV gametocytes had an elongated shape with pointy ends and highly condensed chromatin. On D10, both cultures consisted exclusively of mature stage IV and stage V gametocytes, the latter characterized by their thick, falciform shape with rounded ends. Looking at the number of gametocytes generated per millilitre of erythrocytes used (see **Supplementary Figure 1**), there was a trend for NF54 cultures producing more gametocytes (10.7 ± 5.7 million for iGP1, 19 ± 5.8 million for NF54). We also generated mature iGP1-gametocytes and performed exflagellation experiments to check functionality of male gametocytes. We were able to successfully do so using a temperature drop (see **Supplementary Figure 2**), underpinning iGP1 gametocyte viability and quality. Taken together, this indicates that both iGP1 and NF54 allowed to produce healthy immature and mature gametocytes. However, iGP1 cultures showed a higher stage synchronicity at D4, underscoring the superiority of this strain to produce immature gametocyte populations for subsequent drug sensitivity testing.

### 3.2 Designing a robust microdilution drug sensitivity assay for immature stage II/III iGP1-parasites

Next, we aimed to establish a reliable assay to quantify drug activity profiles against immature gametocytes in a 96-well microdilution format. After an initial round of experiments to determine optimal assay conditions (summarized in the methods section), we decided to continue with 50,000 gametocytes seeded per well, a 48h incubation time with the compounds and measurement of gametocyte viability using the luciferase-based ATP detection kit BacTiter-GLO.

**Figure 5:**
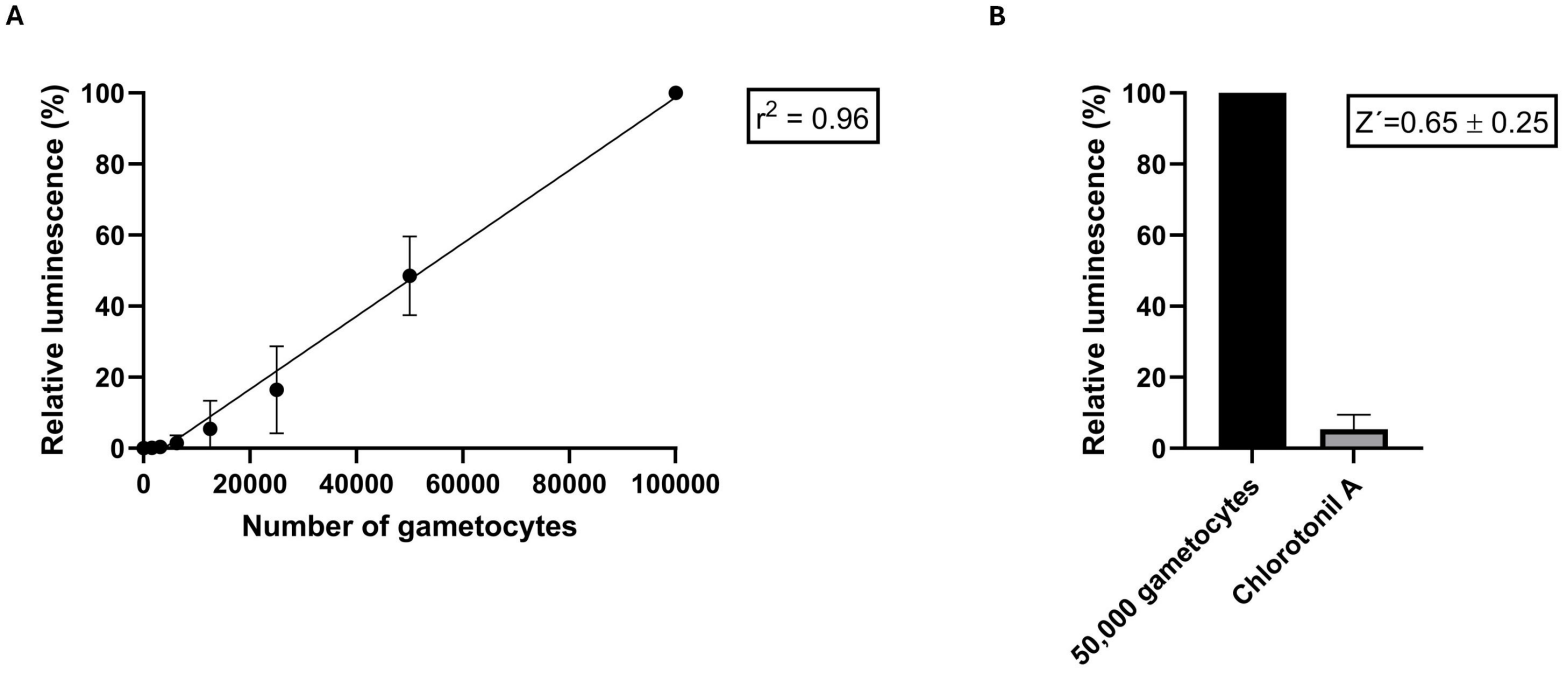
Evaluation of the established drug sensitivity assay using the iGP1 strain. **(A)** The microscopically determined number of immature stage II/III gametocytes shows a strong linear correlation to relative luminescence corresponding to ATP-production with r^2^ = 0.96 (five biological replicates with at least two technical replicates). **(B)** Relative luminescence of 50,000 untreated gametocytes (positive control) and the highest concentration of chlorotonil A (3.75 µM or 4.33 µM, negative control). Z´-factor was calculated separately from absolute luminescence measurements of 5 biological replicates with at least 2 technical replicates.

To determine the reliability and quality of our assay we performed measurements using serial dilutions of purified immature iGP1 gametocytes. First, a simple linear regression of microscopically counted gametocytes and luminescence was performed (**Figure 5A**). We observed a strong linear correlation between the number of immature gametocytes and relative luminescence (r^2^ = 0.96), indicating adequate quantification of viable gametocytes in the tested range. Then, the Z´-factor (indicating the separation of positive and negative control in the dynamic range of the assay) were calculated. 50,000 untreated immature gametocytes, the same number that is later seeded in each well during the drug sensitivity assays, was used as positive control and the highest concentration of chlorotonil A, a potent gametocytocidal (Held et al., 2014), as negative control (**Figure 5B**). The resulting Z´-factor of 0.65 ± 0.25 indicates excellent assay quality (Zhang et al., 1999).

### 3.3 Activity of commonly used and novel antimalarial compounds against immature stage II/III iGP1 gametocytes

**Figure 6:**
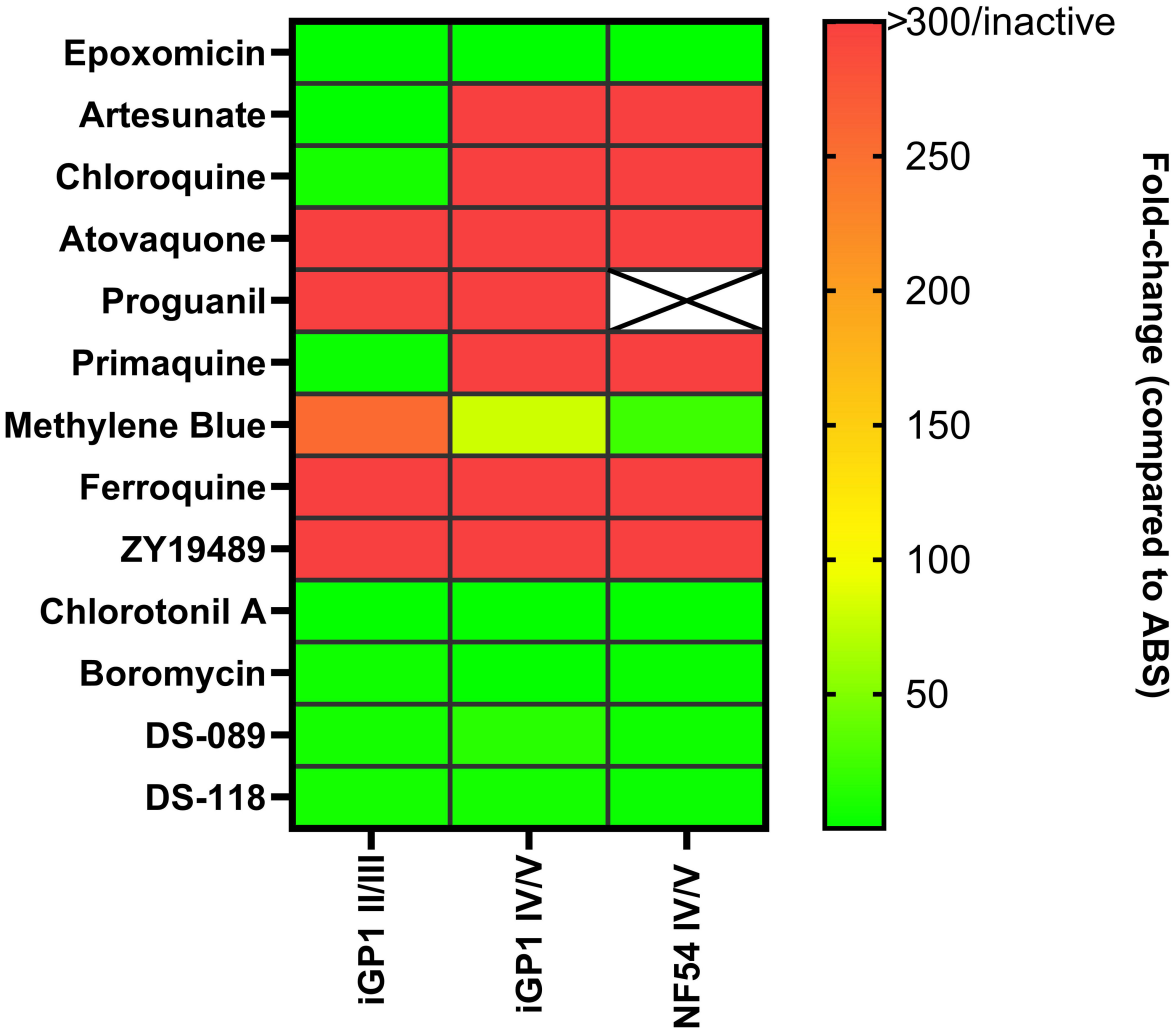
Heat map depicting the fold-change of the IC_50_s of the tested compounds comparing different gametocyte stages (either immature stage II/III or mature stage IV/V) to asexual blood stage parasites of iGP1 and NF54. Green colour indicates potent dual-stage activity, while yellow to orange corresponds to residual activity against gametocytes. Red marks a high IC_50_-shift to complete inactivity against gametocytes. Epoxomicin, methylene blue and the novel compounds chlorotonil A, boromycin, DS-089 and DS-118 proved to be dual-active compounds (with the caveat of low activity of methylene blue against immature stage II/III gametocytes), while artesunate and chloroquine largely retained their activity only against immature stage II/III gametocytes. The other approved antimalarial drugs were selective for asexual blood stages. Since proguanil did not show sufficient activity against asexual NF54 parasites, this comparison was excluded.

Subsequently, we decided to further validate our assay by testing a panel of 13 antimalarial compounds against immature iGP1-gametocytes, including gametocytocidal compounds, commonly used antimalarials as well as novel molecules with previously described antimalarial activity. Epoxomicin and methylene blue, which are both known to exhibit gametocytocidal activity, served as positive controls together with chlorotonil A, an especially potent gametocytocidal. While epoxomicin exhibited strong, single digit nanomolar activity against immature gametocytes as expected, methylene blue only showed weak activity in the low micromolar range. The activity of epoxomicin was even stronger against the immature gametocytes compared to asexual blood stages, while we observed a substantial, 254.5-fold decrease in activity for methylene blue (see **Table 1** for exact values, **Supplementary Figures 3–5** for dose-response curves and **Figure 6** for the fold-change of IC_50_s for immature stage II/III gametocytes compared to asexual blood stages). Among the antimalarials currently in use, only artesunate showed pronounced activity of nanomolar magnitude. While chloroquine showed considerable, sub-micromolar activity, atovaquone and proguanil were inactive at the tested concentrations. Primaquine, one of the few gametocytocidal drugs in use, showed a low activity with an IC_50_ in the single digit micromolar range as expected, as it is in its unmetabolized form. The two novel antimalarial candidates ferroquine and ZY19489, which are currently under clinical development as a new treatment combination, proved to be inactive against immature gametocytes at the tested concentrations.

**Table 1:** Activity of approved and novel antimalarial compounds against immature stage II/III iGP1 gametocytes and iGP1 and NF54 asexual blood stages (ABS).

Compound	iGP1 II/III IC_50_ + SD [nM]	iGP1 II/III IC_90_ + SD [nM]	iGP1 ABS IC_50_ + SD [nM]	NF54 ABS IC_50_ + SD [nM]
Epoxomicin	1.7 ± 1	2.7 ± 1.4	11.2 ± 1.3	10.4 ± 0.3
Methylene blue	2,131.4 ± 968.3	5,157.3 ± 3,901.4°	8.4 ± 1.8	11.3 ± 0.6
Artesunate	4.3 ± 1.6	6.8 ± 0.8	8.5 ± 0.01	17.1 ± 0.9
Chloroquine	86.8 ± 56.7	149.9 ± 119.2	10.7 ± 0.4	13.3 ± 2.2
Primaquine	9,356 ± 9,078.7°	12,928.7 ± 12,307.9°	2,146.9 ± 173.9	2,995 ± 163.7
Atovaquone	> 2,600	> 2,600	0.05 ± 0.01	0.2 ± 0.0002
Proguanil	> 26,000°	> 26,000°	108.6 ± 17.2	> 44,000
Ferroquine	> 300	> 300	0.9 ± 0.2	1.2 ± 0.3
ZY19489	> 300	> 300	8 ± 0.2	9.5 ± 1.5
Chlorotonil A	18.6 ± 4.8	45.4 ± 19.4	13.8 ± 0.5	16 ± 2.8
Boromycin	15 ± 10.1°	30.9 ± 27.2°	2.5 ± 1.7	2.5 ± 0.3
DS-089	526.8 ± 316.7	1,459 ± 1,351.3	66.7 ± 1.1	92.7 ± 3.1
DS-118	1,627.1 ± 1,912.4°	2,211.3 ± 2,568.8	214.6 ± 9.4	251.7 ± 37.1

Viability was determined after 48h incubation with a luciferase-based ATP detection kit. For immature stage II/III gametocytes, values represent 3 biological replicates per data point, except for drugs marked with ° (2 biological replicates). For asexual blood stages, values represent two biological replicates. Dose-response-curves can be found in **Supplementary Figures 3**–**5**.

In addition, we investigated whether four novel antimalarial compounds with previously known activity against asexual blood-stages and mature gametocytes or liver-stage parasites (Held et al., 2014; Stopper et al., 2024) also acted against immature gametocytes. Two natural compounds, namely chlorotonil A and boromycin, showed strong, nanomolar activity against immature gametocytes, which were comparable to activities against asexual stages (see **Table 1**; **Figure 6**). Finally, we also assessed two synthetic histone deacetylase inhibitors (HDACi) termed DS-089 and DS-118. We found a moderate activity with a sub-micromolar IC_50_-values for DS-089 and a considerably lower activity for DS-118 in the micromolar range. Compared to asexual stages, both HDACi showed a moderate shift in activity (< 10-fold). In conclusion, we were able to show that the drug sensitivity assay described above allows accurate activity profiling of chemically diverse compounds with varying levels of activity against immature gametocytes.

### 3.4 Activity of commonly used and novel antimalarial compounds against mature stage IV/V gametocytes

Next, we aimed to extend this approach to mature gametocytes and to compare whether iGP1 mature stage IV/V gametocytes had a similar response to the above-mentioned antimalarial compounds compared to conventionally produced NF54 mature stage IV/V gametocytes (**Table 2**). Concerning the known gametocytocidal control drugs, we observed comparable activity in the single-digit nanomolar range for epoxomicin against both iGP1 and NF54. Methylene blue showed the expected activity with an IC_50_ of roughly 250 nM against NF54 and was roughly two times less active against iGP1. As expected, artesunate, atovaquone and the pro-drug primaquine did not show activity in a relevant concentration against mature gametocytes of both strains. Chloroquine and proguanil also showed no activity or only unspecific toxicity at the highest concentrations against mature stage IV/V NF54 and iGP1 gametocytes. In addition, ferroquine and ZY19489 failed to exhibit notable activity against both strains in the tested concentrations. As previously described, chlorotonil A and boromycin had a pronounced activity against mature stage IV/V gametocytes and at a comparable level for both iGP1 and NF54. Regarding the synthetic HDACi, DS-089 had a submicromolar IC_50_ against NF54 and a roughly doubled IC**_50_** of 1 µM against iGP1, while DS-118 exhibited micromolar activity against both strains. Taken together, mature iGP1 gametocytes showed comparable drug responses compared to conventionally produced NF54 mature stage IV/V gametocytes. In comparison to asexual blood stages, all approved antimalarials showed no activity or only unspecific toxicity at the highest concentrations (see **Table 1**; **Figure 6**). Notably, the IC_50_s of epoxomicin, chlorotonil A and boromycin remained comparable to asexual blood stages, while a moderate decrease (5- to 15-fold) was observed for DS-089 and DS-118, confirming them all as dual-active compounds (see **Table 1**; **Figure 6**). Furthermore, we calculated selectivity indices (SI) for chlorotonil A, boromycin, DS-089 and DS-118 and the comparator drugs epoxomicin and methylene blue from previously published data (see **Table 3**). The SI of the novel compounds chlorotonil A, boromycin and DS-089 were > 200 and >150 for DS-118, highlighting the favourable activity profile of these compounds for the development as antimalarials. Especially boromycin proved to be highly selective with a 6-digit SI.

**Table 2:** Activity of approved and novel antimalarial compounds against iGP1 and mature stage IV/V NF54 gametocytes.

Strain	iGP1			NF54
Compound	IC_50_ + SD [nM]	IC_90_ + SD [nM]	IC_50_ + SD [nM]	IC_90_ + SD [nM]
Epoxomicin	1.1 ± 0.04°	1.3 ± 0.06°	3.2 ± 3.4	8.6 ± 5.3
Artesunate	> 11,000	> 11,000	> 11,000°	> 11,000°
Chloroquine	> 21,000	> 46,000	> 111,000*	> 111,000*
Atovaquone	> 22,000	> 22,000	> 22,000*	> 22,000*
Proguanil	> 111,000	> 111,000	> 18,000*	> 39,000
Primaquine	> 111,000*	> 111,000*	> 111,000*	> 111,000*
Methylene blue	667.9 ± 463.1*	2,669.1 ± 2060.1*	270.4 ± 71.1°	942.6 ± 849.8°
Ferroquine	> 3,000	> 3,000	> 3,000*	> 3,000*
ZY19489	> 4,000	> 6,000	> 4,000*	> 4,000*
Chlorotonil A	16.6 ± 9.2*	146.3 ± 148.8*	19.6 ± 11.8*	32.2 ± 21*
Boromycin	2.7 ± 1.4*	6.5 ± 2.9*	5.8 ± 1.8*	13 ± 4.1*
DS-089	1,048.2 ± 483.2	7,681 ± 7,483.1	467.7 ± 272.3	4,415.8 ± 1,714.5
DS-118	1,620.1 ± 1,528.5	29,201.9 ± 37,162.3	1,170.1 ± 608.1	19,207.8 ± 15,631.7

Viability was determined after 48h incubation with a luciferase-based ATP detection kit. Each data point represents 3 biological replicates, except for values marked with * (4 biological replicates) or ° (2 biological replicates). Dose-response-curves can be found in **Supplementary Figures 6**, **7**. The value of NF54 for methylene blue was measured in the same assay as published in (Reßing et al., 2025) and is used as a shared control.

**Table 3:** Selectivity indices (SI) of highly active novel gametocytocidal compounds for asexual blood stages (ABS), immature stage II/III and mature stage IV/V gametocytes.

Strain	SI iGP1			SI NF54			
Stage	ABS	Immature	Mature	ABS	Mature	Cell line	Citation
Epoxomicin	> 89	> 590.1	> 939.6	> 96	> 310.6	A549	(Czesny et al., 2009)
Methylene blue	320	1.3	4	238	9.9	HepG2	(Fabbri et al., 2023)
Chlorotonil A	> 361.7	> 269.4	> 301.1	> 313.1	> 255.6	HCT-116	(Jungmann et al., 2015)
Boromycin	500,757.7	83,181.2	454,998.9	490,758.7	215,306.1	HepG2	(de Carvalho et al., 2021)
DS-089	> 3,748.5	> 474.6	> 238.5	> 2697.1	> 534.6	HEK293	(Stopper et al., 2024)
DS-118	> 1165	> 153.6	> 154.3	> 993.4	> 213.7	HEK293	(Stopper et al., 2024)

SI were calculated by dividing the cytotoxic concentration 50 (CC_50_) against the human cell line by the IC_50_ of the respective strain and stage. The cytotoxicity data for human cells was obtained from the indicated publications.

## 4 Discussion

To achieve the ambitious goals set forth by the WHO to decrease malaria cases and deaths by 90% in 2030 compared to 2015 (WHO, 2021), strongly reducing parasite transmission in endemic areas is paramount. Therefore, novel antimalarial compounds should ideally exhibit a dual activity against both asexual and sexual blood stages to allow for simultaneous reduction of disease burden and transmission (Burrows et al., 2017). The importance of evaluating compound activities against the long-circulating, transmissible mature gametocytes is increasingly accepted and incorporated into the drug development pipeline due to their essential role in transmission reduction (Katsuno et al., 2015; Burrows et al., 2017; Schäfer et al., 2024). However, the role of immature gametocytes remains often underappreciated, as evidenced by their absence from current screening strategies (Katsuno et al., 2015; Burrows et al., 2017; Schäfer et al., 2024). Incorporating the evaluation of activities against all gametocyte stages by implementing well-functioning, standardized workflows will help to close this gap.

Current methods for culturing gametocytes with subsequent drug sensitivity testing face several difficulties. Firstly, gametocyte culturing techniques are significantly more work- and cost-intensive compared to asexual culture, involving daily medium changes, the use of human serum and a comparably low gametocyte yield (Omorou et al., 2022). Secondly, induction of sexual conversion is not well standardized, as commonly used methods include the use of conditioned medium (Peatey et al., 2011; Ruecker et al., 2014; Brancucci et al., 2015; Plouffe et al., 2016), starvation through reduced medium replacement (Tanaka et al., 2013; Lucantoni et al., 2015), high parasitaemia (Sanders et al., 2014; Gebru et al., 2017) and/or drop in haematocrit (Buchholz et al., 2011; Reader et al., 2022). Some studies sought to increase consistency by using either glucose-free or choline-depleted medium (Reader et al., 2015; Brancucci et al., 2017). Thirdly, protocols that allow the generation of highly synchronous gametocyte populations usually require rigorous synchronization before induction, making the laboratory work even more cumbersome (Brancucci et al., 2015; Plouffe et al., 2016; Reader et al., 2022). One key innovation in the last years addressing these issues was the engineering of the iGP1-strain by Boltryk and colleagues that allow synchronous induction of sexual conversion through coordinated expression of GDV1 (Boltryk et al., 2021). This direct method circumvents biological variation associated with other induction approaches and thus increases reproducibility. In our hands, there was a trend for higher gametocyte yield of NF54 compared to iGP1, which might be attributed to our use of an NF54 strain that is known to be a vigorous gametocyte producer. It has been isolated during a clinical trial from a participant and is expected to yield more gametocytes than NF54 that is only kept in long-term *in vitro* culture (Dixon et al., 2008). Taking into account the comparably low number of replicates and the different starting parasitaemias for iGP1 cultures, this finding points to comparable yields for both strains rather than biologically significant, quantitative differences. While iGP1 gametocyte culture generally involves a similar workload compared to our simplified NF54 protocol, it yielded a more synchronous culture of immature gametocytes, comparable to the roughly 90% purity observed by Boltryk and colleagues (Boltryk et al., 2021). While only a systematic comparison with more replicates would allow a quantitative comparison, this trend still indicates the benefits of the iGP1-strain in this regard. Usually, such a high synchronicity is only achieved with more elaborate protocols that involve especially tight synchronization (Plouffe et al., 2016; Reader et al., 2022), presenting a major advantage of this approach. In addition, we were able to successfully induce exflagellation in iGP1 mature stage IV/V gametocytes, showing functional activity similar to what has been described in literature before (Boltryk et al., 2021).

Furthermore, previously described procedures often use different methods to assess viability of immature stage II/III and mature stage IV/V gametocytes. For the latter, this includes functional assays evaluating cellular morphology, exflagellation and dual gamete formation, an approach that naturally misses immature stages (Ruecker et al., 2014; Lucantoni et al., 2015; Reader et al., 2022). Other methods to directly quantify viable gametocytes include genetically encoded fluorescent proteins or luciferase, resazurin-based or fluorescent dyes as well as luciferase-based techniques to detect ATP (Buchholz et al., 2011; Peatey et al., 2011; Brancucci et al., 2015; Reader et al., 2022). To keep experimental procedures straightforward and comparable for different gametocyte stages, we tested two commercially available kits, namely the viability-specific, resazurin-based dye AlamarBlue and BacTiter-GLO, which detects ATP-levels as a correlate of gametocyte viability. Both approaches have been used previously with mature, but not immature gametocytes for drug sensitivity assays (Tanaka and Williamson, 2011; Lelievre et al., 2012). Since only BacTiter-GLO yielded interpretable results with immature gametocytes, this method was used for all measurements. Our proposed assay has several advantages over currently available methods, namely a reduced workload during cell culture and experimental procedures as well as providing a more standardized pipeline for drug sensitivity testing on both immature and mature gametocytes. We evaluated the robustness of this assay using the Z´-factor, which indicated a well-performing assay of high quality, comparable to a setup previously published for mature gametocytes (Zhang et al., 1999; Lelievre et al., 2012). We could not perform this assay with immature stage II/III NF54 gametocytes, as the low and variable synchronicity of the cultures limited reproducibility and reliability.

We further validated our approach by testing the sensitivity of immature stage II/III iGP1 gametocytes against a panel of chemically diverse compounds consisting of approved antimalarials, known gametocytocidals and novel antiplasmodial molecules. The proteasome inhibitor epoxomicin showed potent activity against immature stage II/III iGP1-gametocytes, comparable to the activity against mature gametocytes determined during this study and in the literature (Held et al., 2014). For methylene blue, we consistently observed an IC_50_ for immature stage II/III iGP1 in the low micromolar range, roughly 50 to 150-fold higher compared to literature values (Plouffe et al., 2016; Reader et al., 2022; Brancucci et al., 2025). To evaluate possible lot-to-lot variability, we also tested a newly acquired batch of methylene blue and several freshly prepared stock solutions. However, this did not influence its activity against immature stage II/III iGP1 gametocytes. We found the expected three-digit nanomolar IC_50_ for methylene blue when using mature stage IV/V NF54 gametocytes as reported by others (Lelievre et al., 2012; Plouffe et al., 2016; Reader et al., 2022), indicating that the increase in IC_50_ is improbable to be related to the detection method. One explanation could be the higher synchronicity of the iGP1 culture, however, recently a study reporting a two-digit nanomolar IC_50_ for immature stage II/III iGP1 gametocytes measured through an encoded luciferase (Brancucci et al., 2025), showing that the difference is unlikely to be caused by strain-specific differences of iGP1. However, this study used a longer incubation time of 72 h, which might increase the activity of methylene blue (Brancucci et al., 2025). Interestingly, a roughly four-fold lower activity of methylene blue against early-stage compared to late-stage gametocytes has previously been observed in *P. falciparum* clinical isolates from India after 48 h incubation, based on morphological evaluation, although no explanation was provided (Wadi et al., 2018). Nevertheless, differences in incubation time alone do not fully explain this discrepancy and warrant further investigation. Artemisinins were reported to be active against immature gametocytes early on (Kumar and Zheng, 1990), and the reduced gametocytaemia associated with artemisinin-based combination therapies (Price et al., 1996; Sinclair et al., 2009) is thought to be mainly due to this early gametocytocidal effect. Consequently, we found an activity in the low nanomolar range for artesunate, comparable to asexual blood-stages and previous studies (Plouffe et al., 2016; Reader et al., 2022). For the 4-aminoquinoline chloroquine, we could determine an IC_50_ similar to what has been reported (roughly 100 nM), likely corresponding to the reduced haemoglobin uptake in immature gametocytes (Hanssen et al., 2012; Lucantoni et al., 2013; Plouffe et al., 2016; Reader et al., 2022). Primaquine, the only WHO-approved drug for malaria transmission reduction, showed a low activity in the single-digit micromolar range against immature gametocytes. This is not surprising, as primaquine is a prodrug that needs to be hydroxylated in the patient’s liver to exert its full effect (Camarda et al., 2019). Atovaquone and proguanil, which are often used as a preventive treatment combination for travellers to malaria-endemic areas (Johnson and Kalra, 2012), did not show any activity against immature gametocytes. This was to be expected, as atovaquone-proguanil is known to reduce transmission to mosquitoes, while not exhibiting a gametocytocidal effect per se (Enosse et al., 2000). Ferroquine and ZY19489 are currently under clinical investigation as a potential non-artemisinin combination therapy (Zydus, 2024). We found that both compounds did not show activity against immature gametocytes at a physiologically relevant concentration, in line with the reported absence of activity against mature gametocytes (MMV, 2026). In the case of chlorotonil A, we found a similar activity against immature stage II/III gametocytes compared to asexuals, thus being active against all plasmodial blood-stages (Held et al., 2014). Even though it has only been used in veterinary medicine, boromycin is known to act against a variety of human pathogens *in vitro*, including viruses, bacteria and asexual stages of *P. falciparum* (Kohno et al., 1996; Moreira et al., 2016; Abenoja et al., 2021; de Carvalho et al., 2021). While there was a roughly 2- to 10-fold shift of IC_50_ compared to asexual blood-stages, boromycin was still highly active, underscoring its great potential for the development as a lead molecule with multi-stage activity. The capacity of HDACi as dual-active antimalarial drugs has been described before (Andrews et al., 2012; Reßing et al., 2025). In our study, we included two synthetic HDACi termed DS-089 and DS-118, for which we saw a roughly 8-fold decrease compared to asexual stages, both remaining moderately active. In general, the IC_50_s obtained for asexual blood-stages of NF54 and iGP1 were slightly higher than those previously obtained for other parasite strains but remain in a comparable range (Stopper et al., 2024).

Finally, we also tested the same panel of compounds against mature stage IV/V iGP1 and NF54 gametocytes. As expected, we saw a comparable activity of epoxomicin for iGP1, NF54 and in the literature (Held et al., 2014). Methylene blue was comparably active against mature stage IV/V gametocytes of iGP1 and NF54, matching previously described values (Lelievre et al., 2012; Plouffe et al., 2016). The activity of artesunate and chloroquine was either radically diminished or absent at the tested concentrations, likely due to drastically decreased haemoglobin uptake and digestion (Hanssen et al., 2012). Atovaquone and ferroquine were again inactive at relevant concentrations, as well as proguanil and ZY19489 which only showed unspecific toxicity at the highest concentrations tested. Chlorotonil A and boromycin retained their potent, nanomolar activity comparable to immature stage II/III iGP1 gametocytes and previous publications (Held et al., 2014; de Carvalho et al., 2021). DS-089 and DS-118 exhibited a markedly decreased activity against mature stage IV/V gametocytes compared to asexuals (roughly 16-fold and 8-fold for iGP1 and 5-fold for NF54, respectively). Akin to what we saw for immature stage II/III gametocytes, DS-089 proved to be the more potent inhibitor. Regarding the novel compounds presented in this study, the combination of “true” dual-stage activity against asexual blood-stages, immature stage II/III and mature stage IV/V gametocytes and high parasite selectivity underpins their potential for the development as novel antimalarial compounds that allow treatment of the symptom-causing asexual blood stages and reduce transmission by efficiently targeting all gametocyte stages.

Recently, Brancucci and colleagues also described an all-in-one pipeline for drug sensitivity assay on different iGP1 gametocyte stages *in vitro* and *in vivo*, mainly differing from our approach by the method to detect parasite viability, namely the expression of a red-shifted luciferase (Brancucci et al., 2025). Robust assay parameters, comparable drug sensitivity profiles as well as the applicability of their approach for *in vivo* studies described therein further underpin the potential of the iGP1-strain to facilitate the development of novel gametocytocidal compounds.

One limitation of this study is the reliance on ATP production as an indicator of gametocyte viability, used as a proxy for measuring the transmission blocking effect of compounds. However, this approach could, for example, miss gametocyte-sterilizing or sporontocidal effects. Standard membrane feeding experiments have shown that low nanomolar concentrations of methylene blue can sterilize mature gametocytes and that atovaquone has a potent sporontocidal effect, both reducing oocyst counts (Enosse et al., 2000; Adjalley et al., 2011; Bradley et al., 2019). In future studies, our approach could therefore be appended by functional methods such as the membrane feeding assays to directly measure a transmission-blocking effect.

In summary, we presented in this study an easy to handle and reliable workflow for drug sensitivity testing against both immature and mature gametocytes using the iGP1-strain. We validated this assay by profiling both antimalarials currently in use as well as experimental antimalarial compounds, including chlorotonil A, boromycin and two synthetic HDACi. The assay described herein can therefore simplify drug sensitivity testing of novel antimalarials, especially against immature stage II/III gametocytes. Thus, it has the potential to expedite the discovery of novel, dual-active antimalarial compounds to design future antimalarial treatments that treat malaria symptoms as well as combat malaria transmission, contributing to achieving the overarching goal of malaria eradication.

## Data Availability

Only the analyzed data in the form of IC50s, dose-response curves, etc. can be found in the manuscript. The raw data is available upon request.
