# Supplementary material for: The inducible gametocyte producer (iGP1) strain is well-suited to produce both immature and mature gametocytes for subsequent drug sensitivity profiling
Source: Front Cell Infect Microbiol. 2026 Jul 1;16:1809263. doi: 10.3389/fcimb.2026.1809263 (PMC13383387; doi:10.3389/fcimb.2026.1809263)
Supplement: Supplementary file 1 [file DataSheet1.docx]

Supplementary Material


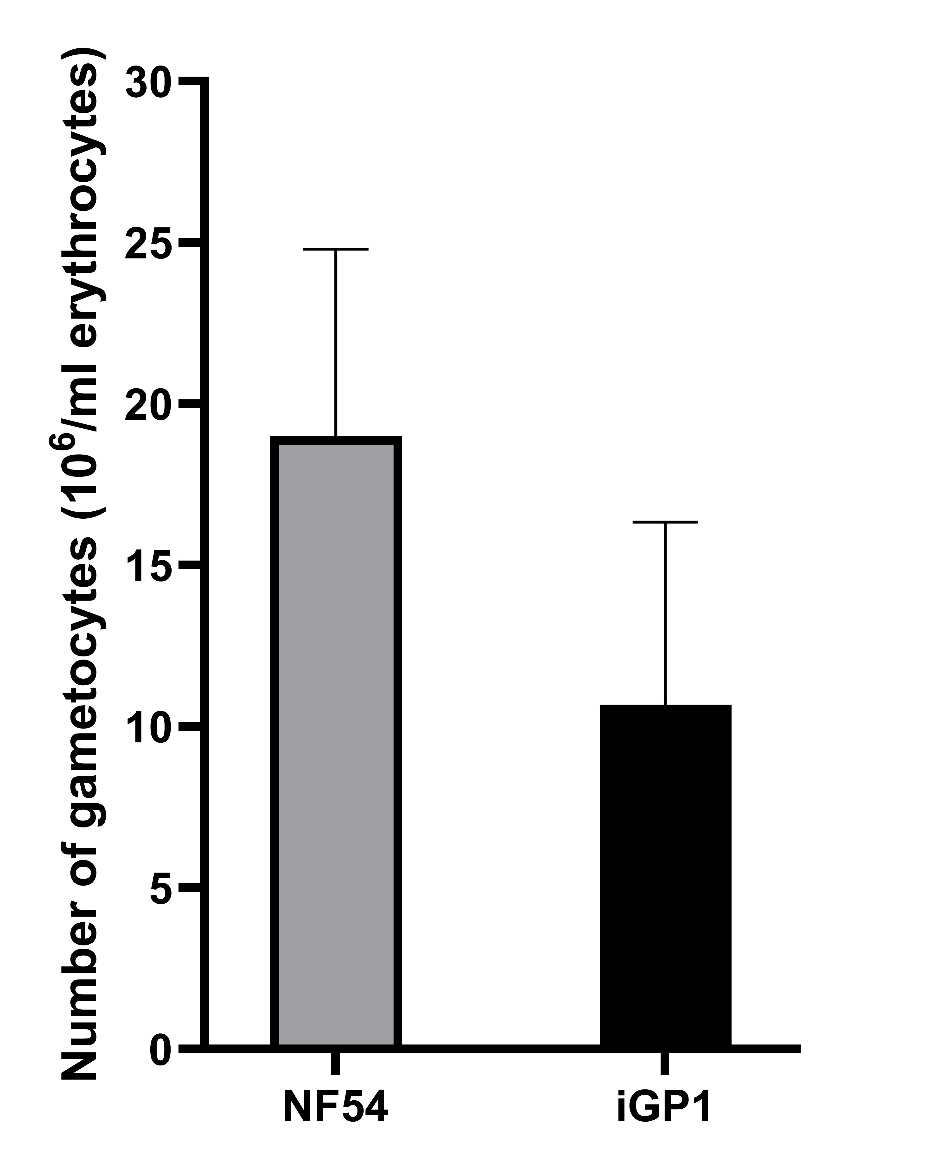


**Supplementary Figure 1.** Comparison of the number of immature gametocytes produced per culture flask of iGP1 (40 ml total volume containing 1 ml erythrocytes) and NF54 (50 ml containing 1.5 ml erythrocytes). Numbers are given in million gametocytes per milliliter of erythrocytes (mean with standard deviation, n = 3 for NF54, n = 5 for iGP1). While there was a trend for a higher gametocyte count for NF54, the difference can partly be explained by the different starting parasitaemias (3% before induction for NF54, 1-3% for iGP1) and the use of an NF54-strain isolated from a patient during a clinical trial (Mordmuller et al., 2015), which influence the final gametocyte density.


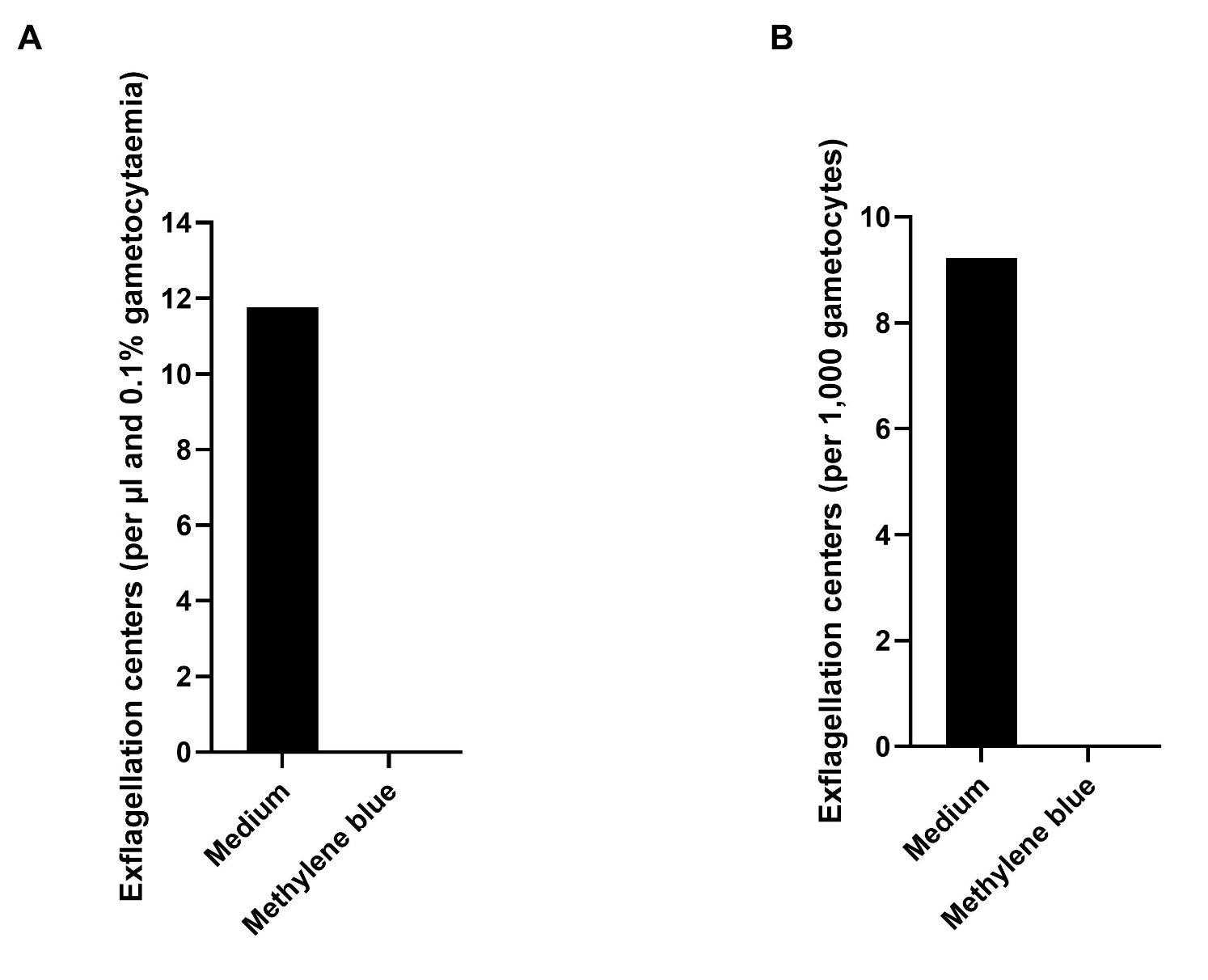


**Supplementary Figure 2.** Comparison of the number of exflagellation centers of mature iGP1-gametocytes incubated with 10 µM methylene blue or medium-only for 48h. Exflagellation centers and erythrocytes were counted in a Neubauer counting chamber and gametocytaemia was quantified using a thin blood smear. A: Exflagellation centers per microliter and 0.1% gametocytaemia of the initial culture. B: Exflagellation centers per 1,000 gametocytes. This graph represents one biological replicate to demonstrate quality of iGP1 gametocytes and does not allow quantification of drug activity.


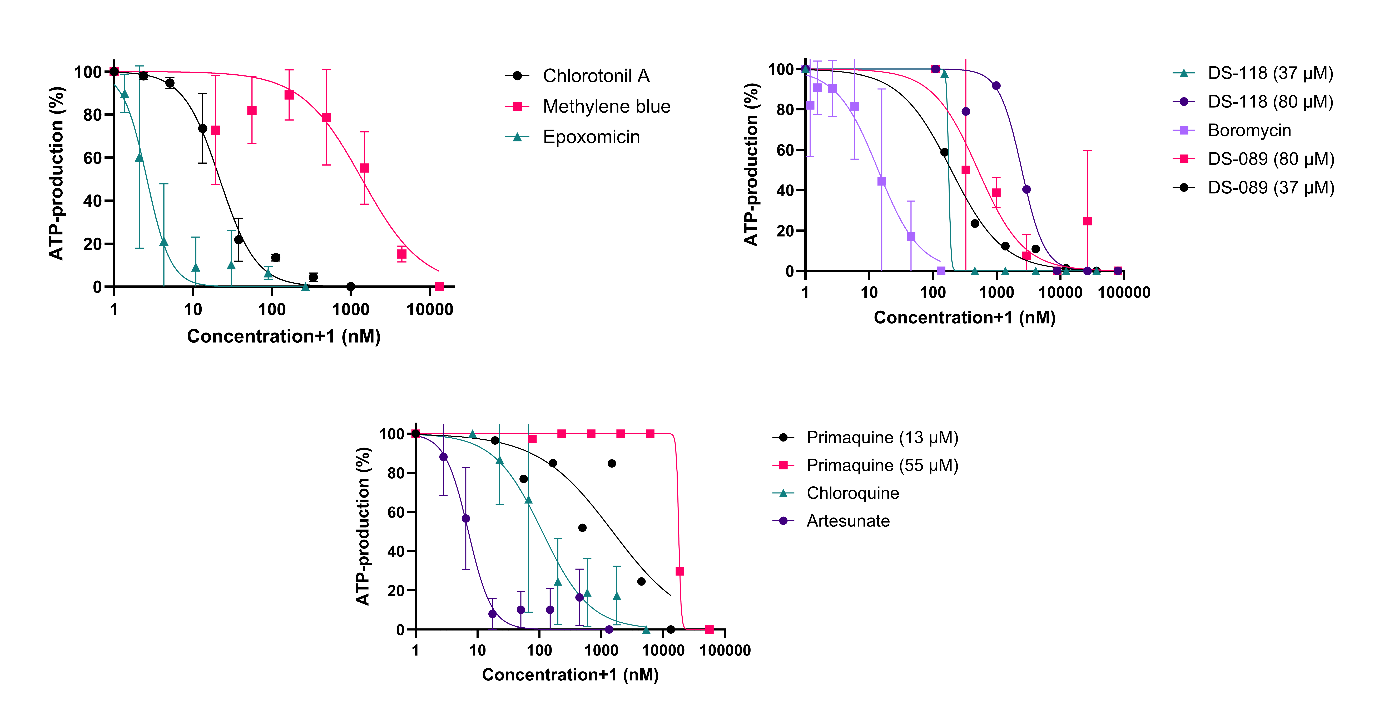


**Supplementary Figure 3.** Dose-response curves for iGP1 immature gametocyte drug sensitivity assays. ATP-production (measured via luminescence) was normalized with the highest concentration of compound as 0% and the drug-free control as 100%. Values below 0% or above 100% were clipped at these points. Data points represent means with SD or in the absence of error bars a single measurement. For compounds with insufficient activity, no dose-response-curve is shown.


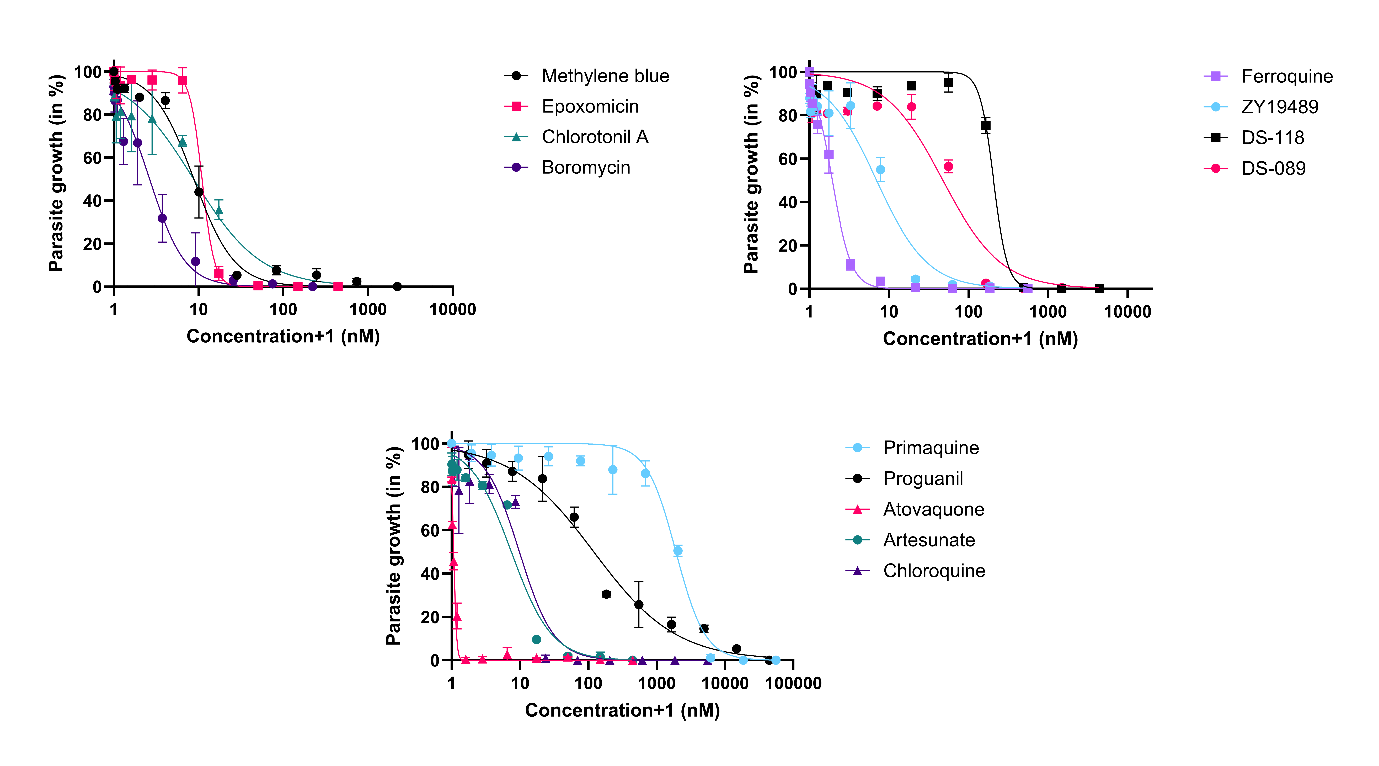


**Supplementary Figure 4.** Dose-response curves for iGP1 asexual blood-stage drug sensitivity assays. Parasite growth (measured by optical density) was normalized with the highest concentration of compound as 0% and the drug-free control as 100%. Values below 0% or above 100% were clipped at these points. Data points represent means with SD. For compounds with insufficient activity, no dose-response-curve is shown.


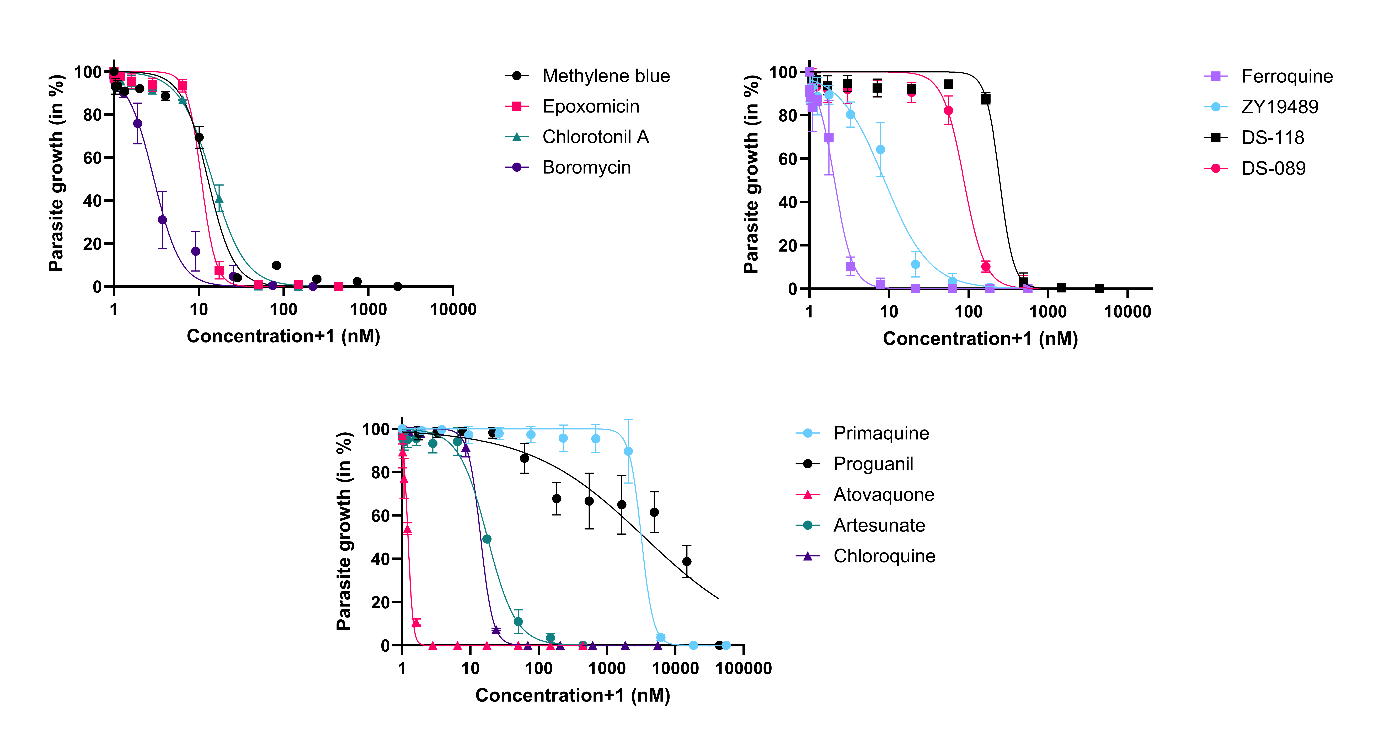


**Supplementary Figure 5.** Dose-response curves for NF54 asexual blood-stage drug sensitivity assays. Parasite growth (measured by optical density) was normalized with the highest concentration of compound as 0% and the drug-free control as 100%. Values below 0% or above 100% were clipped at these points. Data points represent means with SD. For compounds with insufficient activity, no dose-response-curve is shown.


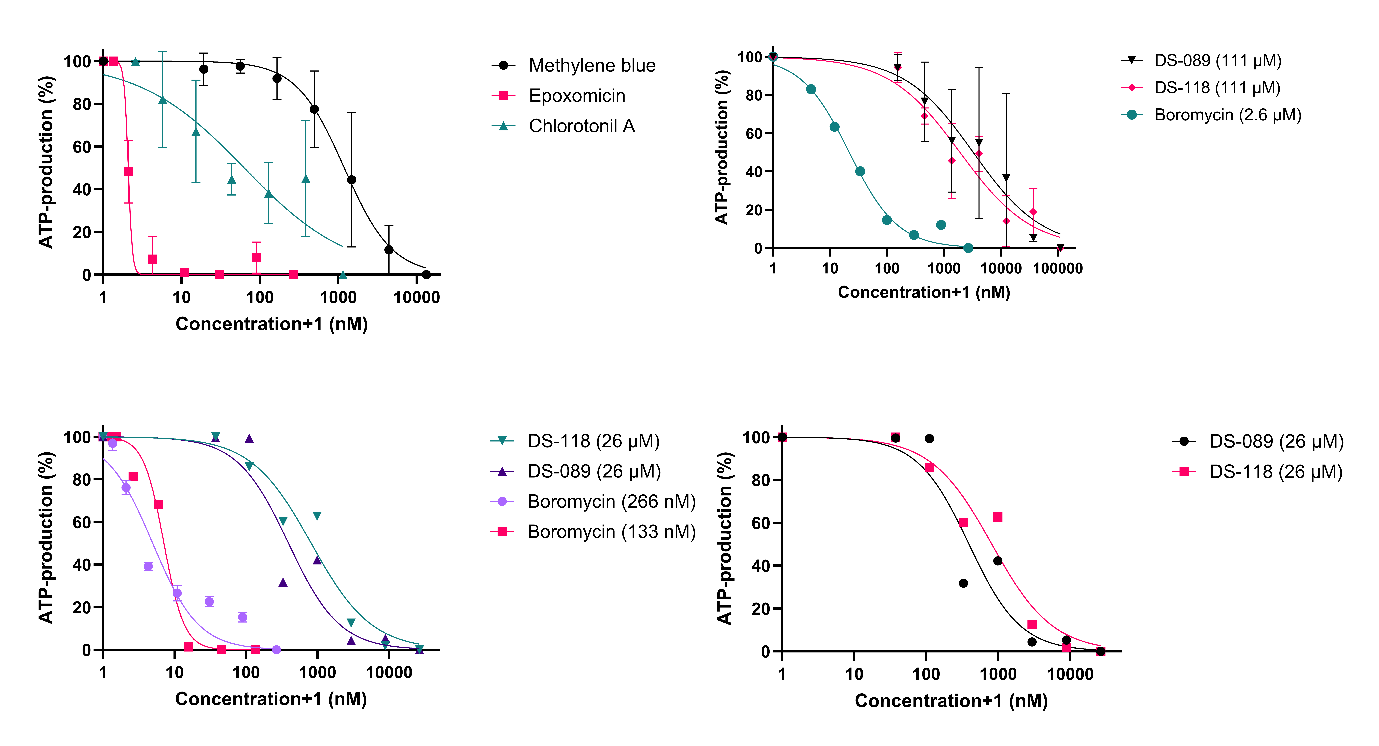


**Supplementary Figure 6.** Dose-response curves for iGP1 mature gametocyte drug sensitivity assays. ATP-production (measured via luminescence) was normalized with the highest concentration of compound as 0% and the drug-free control as 100%. Values below 0% or above 100% were clipped at these points. Data points represent means with SD or in the absence of error bars a single measurement. For compounds with insufficient activity, no dose-response-curve is shown.


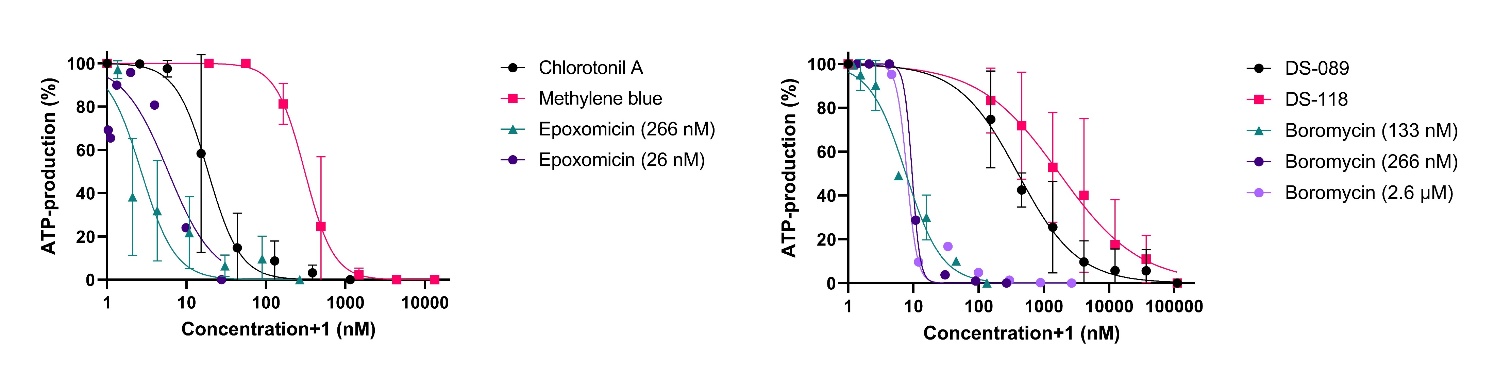


**Supplementary Figure 7.** Dose-response curves for NF54 mature gametocyte drug sensitivity assays. ATP-production (measured via luminescence) was normalized with the highest concentration of compound as 0% and the drug-free control as 100%. Values below 0% or above 100% were clipped at these points. Data points represent means with SD or in the absence of error bars a single measurement. For compounds with insufficient activity, no dose-response-curve is shown.

**Supplementary Table 1.** Concentrations used for drug sensitivity testing. For each drug, 7 dilution steps and one well with drug-free medium were used (two exceptions are noted in the table). The number of biological replicates per concentration is shown by indented letters (a: 1 replicate, b: 2 replicates, c: 3 replicates, d: 4 replicates).

| **Drug** | **iGP1 immature [nM]** | **iGP1 mature [nM]** | **NF54 mature [nM]** | **iGP1/NF54 asexual [nM]** |
| --- | --- | --- | --- | --- |
| Epoxomicin | 266.7-0.4^c^ | 266.7-0.4^b^ | 266.7-0.4^b^; 26.7-0.04^a^ | 444.4-0.008^b^ |
| Methylene blue | 13,333.3-18.3 ^c^ | 13,333.3-18.3^d^ | 13,333.3-18.3^b^ | 2,222.2-0.038^b^ |
| Artesunate | 1,333.3-1.8 ^c^ | 11,111.1-15.2^c^ | 11,111.1-15.2^b^ | 444.4-0.008^b^ |
| Chloroquine | 5333.3-7.3 ^c^ | 111,111.1-152.4^c^ | 111,111.1-152.4^d^ | 5,555.6-0.09^b^ |
| Atovaquone | 2,666.7-3.7 ^c^ | 22,222.2-30.5^c^ | 2,222.2-30.5^d^ | 444.4-0.008^b^ |
| Proguanil | 26,666.7-36.6^b^ | 111,111.1-152.4^c^ | 111,111.1-152.4^d^ | 44,444.4-0.8^b^ |
| Primaquine | 13,333.3-18.3^a^; 55,555.6-76.2^a^ | 13,333.3-18.3^a^; 55,555.6-76.2^b^; 111,111.1-152.4^b^ | 13,333.3-18.3^d^; 55,555.6-76.2^b^; 111,111.1-152.4^b^ | 55,555.5-0.9^b^ |
| ZY19489 | 333.3-0.46^d^ | 11,111.1-15.2^c^ | 11,111.1-15.2^d^ | 555.6-0.009^b^ |
| Ferroquine | 333.3-0.46 ^d^ | 3066.7-4.2^c^ | 3066.7-4.2^d^ | 555.6-0.009^b^ |
| Chlorotonil A | 1,000-1.4 ^c^ | 1554.7-1.6^d^ | 1554.7-1.6^d^ | 444.4-0.008 |
| Boromycin | 133.3-0.2^b^ | 133.3-0.2^a^; 266.7-0.4^b^; 2,666.7-3.7^a^ | 133.3-0.2^b^; 266.7-0.4^a^; 2,666.7-3.7^a^ | 222.2-0.004^b^ |
| DS-089 | 37037-152.4 (only six dilution steps)^a^; 8000-109.7^b^ | 111,111.1-152.4^c^; 26,666.7-36.6^a^ | 111,111.1-152.4^c^ | 4444.4-0.08^b^ |
| DS-118 | 37037-152.4^a^ (only six dilution steps); 8000-109.7^a^ | 111,111.1-152.4^c^; 26,666.7-36.6^a^ | 111,111.1-152.4^c^ | 4444.4-0.08^b^ |

**Bibliography**

Mordmuller, B., Supan, C., Sim, K.L., Gomez-Perez, G.P., Ospina Salazar, C.L., Held, J., et al. (2015). Direct venous inoculation of Plasmodium falciparum sporozoites for controlled human malaria infection: a dose-finding trial in two centres. *Malar J* 14**,** 117. doi: 10.1186/s12936-015-0628-0.
